# On-Field Assessment of Joint Load in Football Using Machine Learning (Part II)

**DOI:** 10.3390/s26082562

**Published:** 2026-04-21

**Authors:** Anne Benjaminse, Margherita Mendicino, Eline M. Nijmeijer, Pietro Margheriti, Alli Gokeler, Stefano Di Paolo

**Affiliations:** 1 Department of Human Movement Sciences, University Medical Center Groningen, University of Groningen, 9713 AV Groningen, The Netherlands; e.m.nijmeijer@umcg.nl; 2 2nd Orthopaedic and Traumatological Clinic, IRCCS Rizzoli Orthopaedic Institute, 40136 Bologna, Italy; margherita.mendicino@ior.it (M.M.); margheritipietro@gmail.com (P.M.); 3 Exercise Science and Neuroscience Unit, Department of Exercise and Health, Paderborn University, 33098 Paderborn, Germany; alli.gokeler@uni-paderborn.de; 4 Education and Research Department, Isokinetic Medical Group, FIFA Medical Centre of Excellence, 40132 Bologna, Italy; s.dipaolo@isokinetic.com

**Keywords:** ACL injury, agility, female youth athletes, sensors, agility, injury risk profiling

## Abstract

Anterior cruciate ligament (ACL) injury risk is elevated in female youth football, yet knee joint loading has mainly been studied under controlled laboratory conditions. This limits understanding of how injury risk emerges during realistic match situations. This study provided a field-based kinetic characterization of football-specific movements by estimating knee abduction moments (KAMs) using wearable sensors and machine learning. Fifty-two highly talented female youth players performed agility tasks during training, including structured exercises (F-EX) and game-based play (F-GAME). Full-body kinematics were collected with inertial measurement units, and a validated support vector machine model, trained on synchronized motion capture and force plate data, classified trials as high or low KAM. Across 662 change-in-direction trials, 9–12% were classified as high KAM in both conditions, indicating that potentially high-risk loading regularly occurs during routine actions. High KAM trials showed reduced knee and pelvis flexion, increased hip flexion, and greater pelvis rotation toward the cutting direction, reflecting upright, stiff movement strategies. Performance analyses revealed smaller cut angles in exercises and greater approach acceleration in game play, without differences in peak velocity. These findings demonstrate the feasibility of field-based kinetic screening and support a complex-systems perspective on ACL injury risk.

## 1. Introduction

Anterior cruciate ligament (ACL) injury risk is elevated in female football, particularly among youth athletes, owing to the sport’s demands for high-intensity movements like cutting and pivoting. Previous studies have emphasized the critical role of biomechanics in understanding ACL injury risk, particularly in laboratory situations [1]. In football, movement emerges not from the player in isolation, but through a continuously evolving interaction between the player’s individual constraints, the affordances of a stimulus-rich environment, and the specific demands of the task [2]. Preserving the integrity of the task–player–environment relationship enhances ecological validity and, in turn, improves the generalizability of findings, marking an important advancement in ACL injury risk research. When testing on the field, players have room for self-organization of their movements [1]. By accounting for how movement emerges in realistic game-like contexts, this integrated perspective provides deeper insight into the true loading experienced by the knee joint during football-specific maneuvers.

In the context of ACL injury, knee joint loading is commonly described through the knee abduction moment (KAM), which has consistently been identified as a clinically relevant surrogate measure and a strong prospective predictor of ACL injuries [3,4]. Although joint loading is inherently multi-dimensional, including flexion–extension and internal–external rotation moments, KAM is used in the literature as the primary metric due to its strong association with ACL injury risk [5,6,7]. Indeed, recent field-based research [8] has further highlighted that KAM is highly sensitive to the complex and unanticipated constraints of real-world football scenarios, supporting its use as a targeted metric for on-field risk evaluation. This includes factors such as team dynamics, training load, and an athlete’s physical and psychosocial characteristics, which is naturally captured. Together, these factors shape an individual player’s risk of ACL injury.

Recent advances in data-driven modeling have further expanded the understanding of ACL injury mechanisms, particularly through the application of machine learning and deep learning techniques. These approaches enable the integration of high-dimensional biomechanical data and have demonstrated strong potential in predicting ligament loading, injury risk, and fatigue failure mechanisms under complex, real-world conditions. For example, a recent deep learning framework combining wearable sensor data with subject-specific musculoskeletal modeling has been shown to accurately predict ligament loading states and fatigue failure risk in real time, overcoming key limitations of traditional experimental and computational approaches [9].

In our earlier work, we systematically compared lab and field-based kinematics in youth elite female football players to highlight the ecological validity of in situ movement assessment [10]. Building on this, we subsequently identified high-risk motion patterns using wearable sensor data combined with advanced data mining approaches [8], and recently developed machine learning models to estimate joint loading from field-based data [11]. These developments align with a broader trend in the literature, where machine learning models are increasingly used to bridge the gap between laboratory-grade biomechanical measurements and scalable, field-based injury risk assessment. In particular, deep learning approaches capable of capturing nonlinear, time-dependent relationships in biomechanical signals have demonstrated improved predictive performance compared to traditional regression-based methods, especially for complex tasks such as estimating internal joint loading and cumulative tissue damage [9].

This manuscript represents the next step in this line of research, with the focus shifted from kinematics (Part I) to kinetics through a descriptive analysis of knee abduction moments (KAMs) during field-based movement tasks. The machine learning model developed in Part I [11], trained on synchronized laboratory motion capture and force plate data, is applied to a large on-field dataset collected using wearable sensors. Through this transition from controlled laboratory conditions to real-world training and competition environments, ecological validity is increased and a more accurate characterization of knee joint loading in high-risk movements is enabled.

The novelty of the study is reflected in the provision of kinetic estimates derived from field-based activities, marking a methodological advance in injury biomechanics. By quantifying joint loads in real-world football scenarios, the gap between laboratory inference and on-field application is addressed. In accordance with the structure established in the initial kinematic study [11], two conditions (F-EX and F-GAME) are examined, and the influences of cutting angle, approach speed, acceleration, and task complexity on estimated KAMs are evaluated. The emergence of high knee joint loads in naturalistic play and their variation across contextual conditions are thereby investigated.

In summary, despite extensive biomechanical research on ACL injury risk, knee joint loading has predominantly been quantified under controlled laboratory conditions, limiting insight into how loads emerge during realistic, game-like football actions. The present study addresses this gap by providing field-based estimates of knee abduction moments during football-specific movements, thereby extending kinetic assessment from laboratory inference to ecologically valid on-field contexts. The machine learning framework established in Part I is extended through its application to field data, resulting in the first detailed kinetic description of knee joint loading during football movements. These findings allow previously identified high-risk motions [8] to be contextualized and add an essential kinetic dimension to ongoing ACL injury risk research.

## 2. Materials and Methods

### 2.1. Participants

All procedures were approved by the Medical Ethical Committee of (ID number: METc 2018.249). Fifty-two healthy highly talented female football (soccer) players (mean age 14.8 ± 0.9 years, height 167.6 ± 4.9 cm, mass 56.8 ± 7.1 kg) were included. All players competed in either the highest or second-highest level in Groningen, the Netherlands, participating in four to five training sessions and one official game per week. Players’ dominant leg was identified as the preferred leg to jump and land with [10]. A total of 42 players were identified as right dominant. All players and their parents/legal guardians signed informed consent before inclusion.

Adequate sample size was determined in a previous study [11], which used similar methodologies, by conducting a priori power analysis in G*Power (Version 3.1.9). The analysis indicated that a minimum of 17 participants was required to achieve a statistical power of 0.80 for a medium effect size (partial η^2^ = 0.10, α = 0.05).

### 2.2. Data Collection

Data collection was performed in regular training sessions during the competitive seasons (September–December and January–May) spanning over the years 2018–2025 (no measurements in season 2021–2022 due to COVID). Prior to data collection, anthropometric measurements were obtained for each player.

Players were equipped with an MVN Lycra suit (Xsens Technologies, Enschede, The Netherlands) incorporating 17 inertial measurement units (IMUs), a battery, and an integrated transmission module. Each IMU was positioned in a designated pocket embedded in the suit at specific anatomical landmarks: the right side of the head, sternum, posterior aspect of the hands, dorsal side of the wrists, lateral side above the elbows, midpoint of the scapular spines, midpoint of both posterior superior iliac spines (pelvis), lateral side of the thighs, medial surface of the tibias, and an additional pocket affixed to the dorsal surface of the forefoot according to manufacturer’s recommendations (https://www.movella.com/xsens, accessed on 16 April 2026). Each IMU contained a 3D magnetometer sampling internally at 1 kHz, with an overall system sampling rate of 240 Hz. A calibration procedure was conducted in accordance with the manufacturer’s recommendations [12].

**Figure 1:**
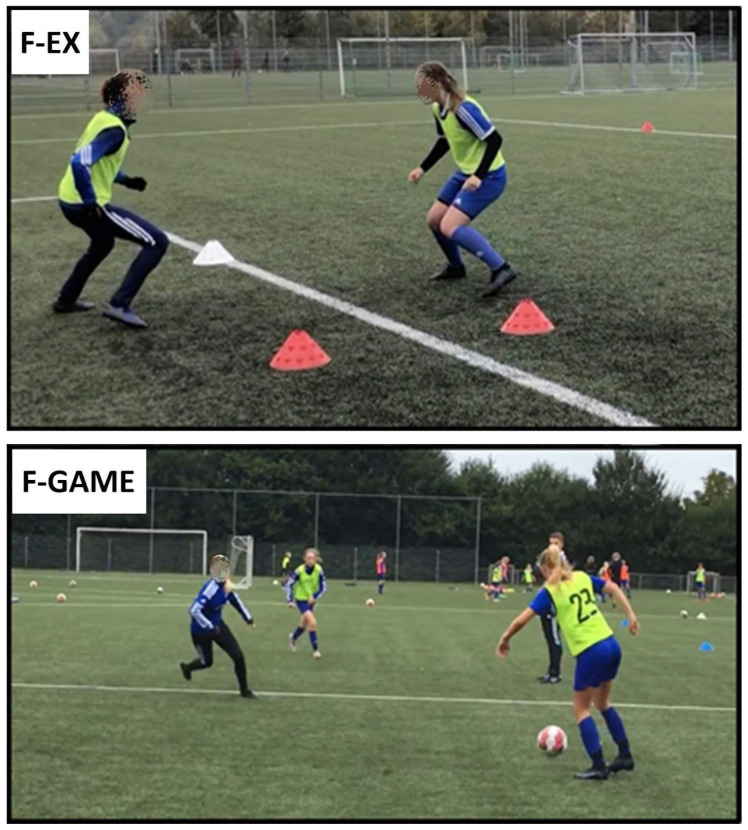
Data collection setting: exercise training condition (F-EX) on the top panel (example: warm-up drill) and game condition (F-GAME) on the bottom panel (example: defensive action). Adapted from [10].

A detailed description of data collection is provided in our previous study [10]. In brief, an entire training session (75 min) was recorded per player and all players included in the study performed the on-field tasks earlier described [10]. Cutting tasks included agility movements that were divided into two conditions: exercise (F-EX) and game (F-GAME) (Figure 1). The F-EX covered different football-specific exercises, including in particular the change in direction tasks. Unexpected elements such as the presence of the ball and/or an opponent were always included (e.g., performing a change in direction reaction to a ball or opponent). The F-GAME instead consisted of a training match performed at the end of the training session [10].

### 2.3. Data Processing

Data collected from all trials were first extracted from the Xsens suit proprietary software, Xsens MVN Analyze 2020.0.1 (Xsens Technologies, Enschede, The Netherlands), and processed in a custom MATLAB R2024a script (The MathWorks, Natick, MA, USA).

Concerning the kinematics analysis, for each recorded training session, all changes in direction performed with the dominant limb were isolated through visual inspection of a single experienced operator [10]. The foot contact window corresponding to the ultimate foot contact before the change in direction [13] was extracted through manual cropping and data were processed in a time-normalized interval from initial contact (0%) to toe-off (100%) [13,14]. Within this temporal window, joint angles were extracted for the ankle, knee, hip and pelvis in the three anatomical planes, for a total of twelve kinematic parameters. The joint coordinate system adhered to the recommendations of the International Society of Biomechanics (ISB), wherein positive rotations are defined as flexion, abduction, and internal rotation [15].

The foot contact window was further segmented into two temporal phases: an entry phase defined from the initial contact to maximum knee flexion; an exit phase defined from maximum knee flexion to toe-off. In line with the previous literature on cutting maneuvers [16], these phases were intended to reflect a load acceptance phase, characterized by braking and energy absorption demands in approaching the change in direction, and a propulsion phase, characterized by force and impulse generation for push-off. Performance analysis focused on extracting the peak values of the tri-axial resultant velocity and acceleration of the center of mass, computed separately for the entry and exit phases of the defined foot contact window, in order to assess approach and push-off performance. Prior to peak acceleration detection, acceleration data were filtered using a fourth-order low-pass Butterworth filter with a 6 Hz cutoff to minimize high-frequency noise and enhance signal quality. In addition, cut angles were calculated over the entire foot contact window and derived from the trajectory of the center of mass using its spatial coordinates.

### 2.4. Machine Learning Knee Abduction Moment Estimation Algorithm

In Part I [11], a Fine Gaussian SVM classification model was developed in MATLAB (Classification Learner App, vR2022a; MathWorks, Natick, MA, USA) to discriminate sidestep cutting maneuvers with high or low KAM. Initially, 26 different regression models were tested to predict continuous KAM values; however, due to suboptimal performance, a classification approach was preferred, with 32 different models evaluated before the ultimate selection of the Fine Gaussian SVM.

The model was trained on data from 32 talented female football players (age 14.8 ± 1.0 y, height 167.9 ± 5.1 cm, mass 57.8 ± 8.0 kg) performing unplanned 45° sidestep cutting maneuvers in response to an opponent in a controlled laboratory setting. Kinematics were captured using Xsens IMUs (Xsens Technologies, Enschede, The Netherlands) synchronized with Vicon motion capture system (Vicon Motion System, Inc., Centennial, CO, USA) and Bertec force plates (Bertec Corporation, Colombus, OH, USA). Classification labels were derived from body-mass normalized peak KAM values. To ensure a clear separation of distinct loading profiles, trials were dichotomized into “high KAM” (+1, >67th percentile) and “low KAM” (−1, <33rd percentile), with the intermediate tertile excluded from the analysis.

The model takes as input the full-body joint kinematics from IMUs: specifically, 12 independent predictors corresponding to the 4 joints (ankle, knee, hip, and pelvis) and 3 anatomical planes. Each predictor represents the joint angle measured at the frame corresponding to the peak KAM, which occurred on average at 7.4 ± 4.7% of the stance phase of the cutting limb [11]. A 5-fold cross-validation was used, reserving a randomly selected 20% of the dataset for testing to reduce overfitting.

As reported in the previous study [11], the model achieved an overall accuracy of 77.9%, with an area under the curve (AUC) of 84.0%, a positive predictive value (PVV) of 79.1%, a sensitivity (true positive rate, TPR) of 72.6%, a specificity (true negative rate, TNR) of 81%, and Youden’s index equal to 0.54. The algorithm is designed to identify high knee joint loads during football players’ agility tasks collected on the field, with the ultimate goal to inform ACL injury prevention strategies.

### 2.5. Statistical Analysis

Data were presented as mean ± standard deviation separately for F-EX and F-GAME conditions. The count and percentage of the tasks classified as high and low KAM was computed. Statistical comparisons were performed through an independent Student’s *t*-test to evaluate whether kinematic and performance metrics differed between high and low KAM groups. Data were considered statistically significant for *p* < 0.05. Mean difference (Δ), *p*-values and Cohen’s *d* effect sizes were reported. Effect size was considered trivial, small, moderate, or excellent if *d* < 0.2, 0.2–0.5, 0.5–0.8, or >0.8, respectively. All analyses were performed in MATLAB R2024a (The MathWorks, Natick, MA, USA).

## 3. Results

A total of 662 change-in-direction trials were collected and analyzed across the 52 participants. Of these, 442 trials corresponded to the F-EX condition, while 220 to the F-GAME condition. The classification model identified trials with high KAM across both experimental conditions, with 54 trials (12.2%) classified as high KAM in F-EX and 20 trials (9.1%) in F-GAME conditions.

### 3.1. Kinematic Difference Between High and Low KAM

Flexion–extension angles at all joints differed significantly between the two groups, in both the F-EX and F-GAME conditions. Specifically, the high KAM group exhibited lower knee flexion (*p* < 0.001, *d* = moderate effect, Table 1) and pelvis anteversion (*p* < 0.001, *d* = moderate-to-excellent effect), but greater hip flexion (*p* < 0.001/*p* < 0.004, *d* = moderate effect) than the low KAM group. Only the F-EX the high KAM group demonstrated lower ankle dorsiflexion (*p* = 0.005, small effect).

In the frontal plane, significant differences were observed only in the F-GAME condition with greater ankle inversion (*p* = 0.042, *d* = small effect) in the high KAM group.

In the transverse plane, consistent patterns emerged across F-EX and F-GAME: the high KAM group showed greater pelvis ipsilateral rotation (*p* < 0.001/*p* < 0.008, *d* = small-to-moderate effect) than the low KAM group.

**Figure 2:**
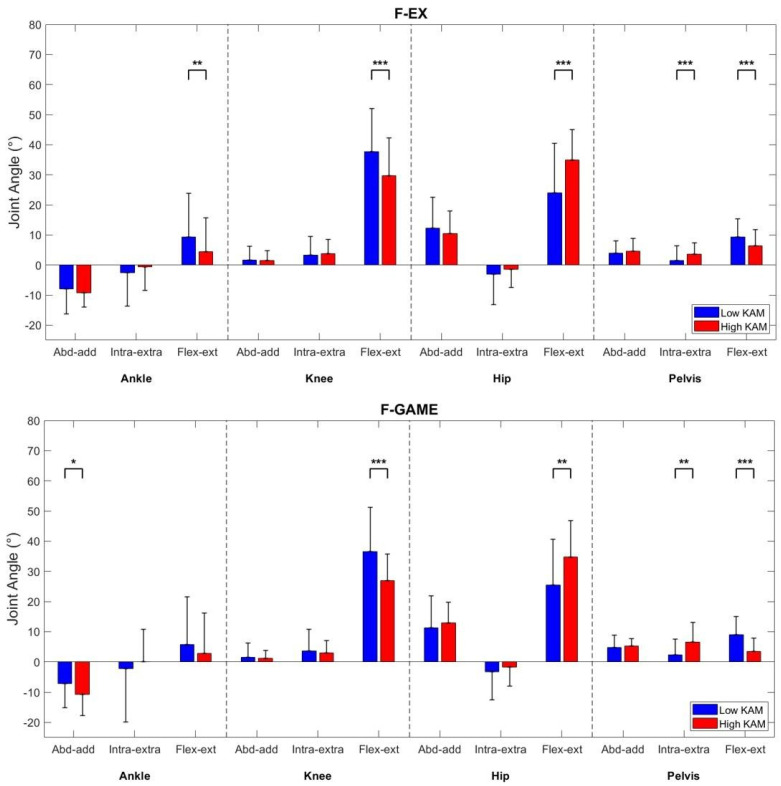
Kinematic differences between high KAM (red) and low KAM (blue) groups in both F-EX and F-GAME conditions. Asterisks denote statistical significance: one (*) for *p* < 0.05, two (**) for *p* < 0.01, and three (***) for *p* < 0.001.

**Figure 3:**
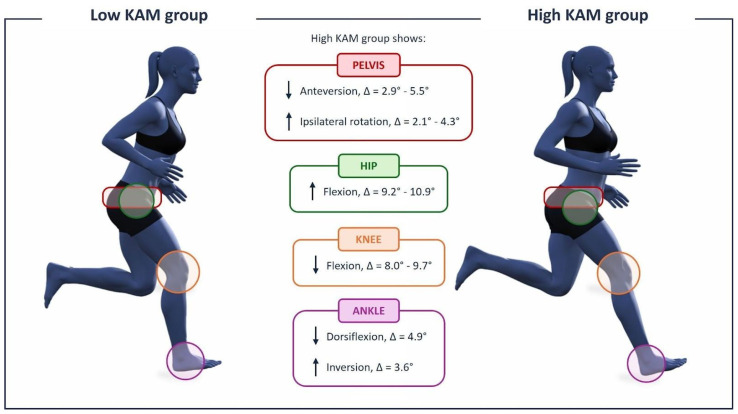
Significant kinematic differences (*p* < 0.05) between low KAM (**left**) and high KAM (**right**) groups, summarized across both F-EX and F-GAME conditions. Arrows indicate whether the high KAM group had higher or lower values than the low KAM group.

All kinematic differences between the two groups are summarized in Figure 2, while Figure 3 graphically displays only the statistically significant differences (*p* < 0.05), emphasizing the main findings.

### 3.2. Performance Between High and Low KAM

The high KAM group showed lower mean cut angle compared to the low KAM group in the F-EX condition (*p* = 0.013, small effect, Table 2), while it demonstrated greater mean peak acceleration in the entry window in the F-GAME condition (*p* = 0.040, moderate effect). No significant differences in mean peak velocity were observed between the high and low KAM groups in either condition (*p* > 0.05).

## 4. Discussion

A central observation of this study is that high KAM trials were consistently associated with distinct kinematic patterns. Across both F-EX and F-GAME conditions, approximately 9–12% of agility trials were classified as high KAM, indicating that potentially high-risk knee loading patterns are not rare anomalies but occur regularly during routine football activities. The similar prevalence across conditions suggests that even relatively structured field exercises can elicit substantial joint loading in a meaningful subset of movements. Given the stochastic and emergent nature of movement in sport, these findings reinforce the notion that ACL injury risk is distributed across common moments of play rather than confined to extreme or visually obvious actions. Across both conditions, players in the high KAM group exhibited reduced knee flexion, reduced pelvis flexion (anteversion), increased hip flexion, and greater pelvis rotation toward the cutting direction, confirming that upright, stiff, and rotated postures increase frontal-plane knee loading during unconstrained field-based movements. Biomechanically, reduced knee flexion during cutting limits sagittal-plane force attenuation by the quadriceps and shifts the contribution of the ground reaction force toward the frontal plane, thereby increasing the external knee abduction moment. In addition, upright trunk and pelvis postures combined with rotation toward the cutting direction displace the body’s center of mass laterally relative to the knee joint center, increasing the frontal-plane moment arm of the ground reaction force and consequently amplifying valgus loading at the knee [17,18,19]. While prior research on high KAM ACL injury mechanisms in women’s football has relied largely on video-based analyses [13,14,20,21], the present study is the first to provide a detailed kinematic characterization of high KAM movements derived from biomechanical data using a machine learning-based approach.

Elevated KAM reflects underlying movement patterns, such as reduced knee flexion, upright trunk posture, and rotational mechanics, that are often associated with suboptimal neuromuscular control during change-in-direction tasks. We further emphasize that identifying these movement configurations in field-based settings may provide actionable information for practitioners, enabling them to target modifiable movement behaviors during agility and neuromuscular training. The robustness of these patterns across both field conditions is noteworthy. It suggests that, despite the self-organizing nature of movement in complex environments, certain movement configurations are reliably associated with elevated joint loads. This supports a complex-systems perspective: athletes may select from a range of movement solutions, but some solutions, whether due to perceptual demands, physical capacity, or decision-making, carry inherently higher biomechanical costs. The observation that high KAM trials were already present during the F-EX condition, and were comparable in magnitude to those recorded in the subsequent F-GAME condition, highlights the effectiveness of this warm-up format in eliciting game-like biomechanical demands. In other words, the F-EX warm-up successfully exposes athletes to the kinds of frontal-plane knee loading patterns they will encounter during actual game play. This underscores its value as a preparatory tool: it not only elevates physiological readiness but also familiarizes athletes with the biomechanical stresses characteristic of competitive situations, potentially improving neuromuscular readiness and reducing the mismatch between traditional warm-up and real-game movement demands [22]. Given this fact, the exercises as in F-EX may also better serve coaches’ desire to have football-specific playing actions in the warming up [22].

Smaller condition-specific differences (e.g., reduced ankle dorsiflexion in high KAM only in F-EX, increased ankle inversion in high KAM only in F-GAME) imply that task and environmental constraints subtly shape the distal strategy used to regulate load. These nuances underscore the value of in situ monitoring, as such interaction-driven variations would be difficult to detect in controlled laboratory tasks.

### 4.1. Performance

The performance findings provide additional ecological context to the kinetic results. In the F-EX condition, high KAM trials were executed with smaller cut angles compared to the low KAM trials, suggesting that sharper direction changes may promote stiffer, higher-load strategies, possibly due to reduced time for preparatory adjustments or increased urgency to maintain speed. Conversely, in the F-GAME condition, high KAM trials were preceded by greater approach-phase acceleration compared to the low KAM trials, indicating that situational game demands may drive athletes into high-load maneuvers not because of the cut itself, but because of the momentum established beforehand.

Interestingly, no differences were detected in peak velocity, reinforcing the idea that how athletes enter a movement (e.g., acceleration profile, timing, anticipation) [23] may be more influential for joint loading than instantaneous speed alone. This aligns with the ecological constraints theory, where perceptual and task context, not isolated physical outputs, shape movement organization [24].

### 4.2. Practical Implications

Together, these findings demonstrate that kinetic screening on the field is not only feasible but also highly informative when ACL injury risk is studied within the complex realities of football performance. By capturing joint loading during authentic, athlete-driven movements that inherently involve task complexity, environmental variability, and opponent-related constraints, this approach moves ACL risk evaluation closer to the conditions under which injuries actually occur [1]. Importantly, the use of wearable sensors enables continuous, in situ measurement across large numbers of trials, naturally incorporating variability in movement strategies that are typically constrained in laboratory settings. While machine learning-based estimation introduces uncertainty related to prediction accuracy, its validated performance allows reliable identification of high-load events without reliance on force plates or optical motion capture, representing a major methodological advance toward scalable and ecologically valid monitoring.

From a practical perspective, the results suggest that screening should extend beyond identifying “risky” athletes and toward identifying “risky” movements within an athlete’s movement repertoire. Given that high KAM emerges in approximately one in ten field actions, practitioners should consider how often athletes adopt stiff, upright, and rotated postures and under which contextual pressures, such as speed demands, decision-making, or opponent interaction, these patterns arise. This shift emphasizes understanding movement variability and constraint interactions rather than static technique deficits. Accordingly, interventions may benefit from targeting movement adaptability, perceptual–motor skill, and preparation strategies instead of enforcing singular “ideal” movement solutions.

Earlier work has shown that athletes exhibiting high KAM during controlled tasks such as drop vertical jumps display distinct whole-body kinematic patterns, heightened responsiveness to targeted interventions, and more deterministic electrocortical activity, particularly within frontal theta and central/parietal alpha-2 frequency bands [25]. These neural signatures have been interpreted as reflecting altered attentional control and sensorimotor processing, factors that may influence how movement is regulated under pressure. The present field-based findings extend this perspective by demonstrating that such high-load movement patterns also emerge in complex, dynamic contexts, where neurocognitive demands and environmental uncertainty are elevated.

Together, these insights underscore the need for ACL injury prevention programs that address both on-field biomechanical control and the neural systems that support adaptive movement behavior. Motor learning strategies such as implicit learning, random and differential practice, and variable, sport-specific tasks that preserve task realism may enhance adaptability and promote resilient perception–action coupling [26]. In line with the current findings, effective prevention should therefore aim to increase flexibility in movement solutions across contexts, rather than impose rigid movement templates that may fail under real-world constraints.

### 4.3. Future Directions

This study further supports a shift away from linear cause–effect models of injury toward a complex-systems framework [27]. The observed kinematic and performance differences between high- and low-load trials demonstrate that joint loading emerges from a constellation of interacting constraints, including approach behavior, interactions with opponents, space–time pressures, individual movement tendencies, and potentially underlying neural processes. By assessing peak knee abduction joint load, a key biomechanical marker associated with ACL injury risk, under realistic field conditions, the present findings capture injury-related mechanisms in a manner that closely reflects actual gameplay demands. This field-based approach inherently incorporates elements of a “web of determinants” [27], enabling a more comprehensive understanding of ACL injury risk than traditional laboratory studies and advancing injury prevention research beyond linear models toward a truly integrative perspective.

While the machine learning model provides a powerful tool for estimating KAM on the field, it is still an approximation trained on laboratory data [8,10,11]. Additionally, longitudinal tracking of load patterns may clarify whether athletes who frequently produce high KAM are indeed at elevated injury risk, a crucial next step for validating this method as a screening tool.

In this context, an additional consideration is the dichotomous classification of KAM, which simplifies an inherently continuous variable to a binary outcome. Although this classification framework demonstrated strong performance [11], aligning with the existing literature and facilitating interpretation, KAM is more appropriately conceptualized along a continuum. Therefore, future work should aim to identify meaningful ranges of KAM exposure rather than relying on a single cut-off value, which may provide a more nuanced and ecologically valid characterization of ACL injury risk.

A further methodological consideration is the predictive variance inherent to the machine learning algorithm. Given the classification accuracy of 77.9% [11], applying the model to field-based data introduces a non-negligible probability of misclassification. The model’s specificity (true negative rate, TNR = 0.81) and sensitivity (true positive rate, TPR = 0.73) indicate that most high and low KAM trials are correctly identified, but some true high KAM movements may be missed, and a few low KAM trials may be erroneously labeled as high. Consequently, this could attenuate the observed differences between high and low KAM trials, potentially biasing the associations between kinematics, performance, and high knee joint loading patterns. Future iterations of this approach should incorporate probabilistic modeling or report statistical confidence intervals to quantify the reliability of automated field-based screening tools.

Lastly, although data were collected in a real-world training environment, the presence of inertial sensor suits and the athletes’ awareness of being monitored may have influenced their natural movement behavior. However, based on informal feedback from the participants, the athletes reported that wearing the sensor suit did not significantly affect their movement behavior, suggesting that the measurement setup was well tolerated and did not meaningfully interfere with typical football movements. Future research may further minimize potential measurement interference by employing emerging markerless motion capture technologies, which enable biomechanical analysis without wearable instrumentation and may allow movement behavior to be assessed even more unobtrusively in natural training and match environments.

## 5. Conclusions

This study demonstrates that elevated knee abduction moments occur regularly during routine football actions and are consistently associated with identifiable kinematic and performance characteristics across different field-based contexts. Upright, stiff, and rotated movement configurations systematically contribute to higher knee joint loading during football-specific actions. High joint loading is not restricted to extreme or rare events but emerges from common movement solutions shaped by task demands, approach behavior, and environmental constraints. By extending ACL injury risk assessment from laboratory-based inference to ecologically valid, field-based conditions, this work provides the first detailed kinetic characterization of football-specific movements using wearable sensors and machine learning. The findings support a complex-systems perspective in which ACL injury risk arises from the interaction of biomechanical, perceptual–cognitive, and contextual factors rather than isolated technique deficits. Practically, this shifts the focus of screening and prevention toward identifying high-risk movement patterns within athletes and developing adaptable, context-sensitive training strategies that better reflect the realities of football performance. Future research should investigate whether the identified high-loading movement patterns can be prospectively used to determine ACL injury risk profiles through longitudinal monitoring across training and competition. In addition, further development of wearable sensor-based models and the integration of these approaches into targeted neuromuscular training interventions may support more individualized and context-specific injury prevention strategies.

## Figures and Tables

**Table 1:** Comparison of full-body kinematics between high KAM and low KAM groups, for both on-F-EX (F-EX) and on-F-GAME (F-GAME) conditions. Joint angles are presented as mean ± standard deviation (in degrees) for both groups, along with the between-group difference (Δ), *p*-value, and Cohen’s *d* effect size. Positive rotations are defined as flexion, abduction, and internal rotation. Positive Δ values indicate higher angles in the high KAM group.

Joint	F-EX					F-GAME				
	High	Low	Δ (%)	*p*-Value	*d*	High	Low	Δ (%)	*p*-Value	*d*
Ankle										
Abd–add	−9.2 ± 4.8	−7.9 ± 8.3	−1.3 (−16.5%)	0.107	0.16	−10.7 ± 7.1	−7.1 ± 8.1	−3.6 (−50.7%)	0.042	0.45
Intra–extra	−0.7 ± 7.7	−2.5 ± 11.1	1.8 (72.0%)	0.143	0.17	0.1 ± 10.7	−2.3 ± 17.5	2.4 (104.3%)	0.364	0.14
Flex–ext	4.4 ± 11.2	9.3 ± 14.5	−4.9 (−52.7%)	0.005	0.35	2.8 ± 13.4	5.7 ± 15.8	−2.9 (−50.9%)	0.369	0.19
Knee										
Abd–add	1.5 ± 3.3	1.6 ± 4.7	−0.1 (−6.3%)	0.886	0.02	1.1 ± 2.7	1.5 ± 4.8	−0.4 (−26.7%)	0.544	0.09
Intra–extra	3.8 ± 4.7	3.3 ± 6.2	0.5 (15.2%)	0.498	0.08	3.0 ± 4.1	3.6 ± 7.2	−0.6 (−16.7%)	0.581	0.09
Flex–ext	29.7 ± 12.5	37.7 ± 14.3	−8.0 (−21.2%)	<0.001	0.57	26.9 ± 8.8	36.6 ± 14.6	−9.7 (−26.5%)	<0.001	0.68
Hip										
Abd–add	10.5 ± 7.4	12.3 ± 10.2	−1.8 (−14.6%)	0.113	0.18	12.9 ± 6.9	11.3 ± 10.5	1.6 (14.2%)	0.356	0.16
Intra–extra	−1.5 ± 5.9	−3.1 ± 10.0	1.6 (51.6%)	0.091	0.17	−1.7 ± 6.3	−3.2 ± 9.4	1.5 (46.9%)	0.345	0.16
Flex–ext	34.9 ± 10.2	24.0 ± 16.4	10.9 (45.4%)	<0.001	0.69	34.7 ± 12.2	25.5 ± 15.2	9.2 (36.1%)	0.004	0.61
Pelvis										
Abd–add	4.6 ± 4.3	3.9 ± 4.1	0.7 (17.9%)	0.294	0.17	5.3 ± 2.4	4.7 ± 4.2	0.6 (12.8%)	0.354	0.15
Intra–extra	3.6 ± 3.7	1.5 ± 4.9	2.1 (140.0%)	<0.001	0.44	6.6 ± 6.5	2.3 ± 5.3	4.3 (187.0%)	0.008	0.80
Flex–ext	6.4 ± 5.4	9.3 ± 6.0	−2.9 (−31.2%)	<0.001	0.49	3.5 ± 4.3	9.0 ± 6.0	−5.5 (−61.1%)	<0.001	0.94

**Table 2:** Comparison of performance metrics between high KAM and low KAM groups, for both on-F-EX (F-EX) and on-F-GAME (F-GAME) conditions. Peak velocity and acceleration are reported separately for foot contact’s entry (IN) and exit (OUT) windows. Values are presented as mean ± standard deviation for both groups, along with the between-group difference (Δ), *p*-value, and Cohen’s *d* effect size.

	F-EX					F-GAME				
	High	Low	Δ (%)	*p*-Value	*d*	High	Low	Δ (%)	*p*-Value	*d*
Peak Velocity IN (m/s)	2.9 ± 0.9	2.8 ± 4.6	0.2 (6.5%)	0.777	0.05	2.8 ± 0.9	2.6 ± 1.5	0.2 (7.7%)	0.568	0.16
Peak Velocity OUT (m/s)	3.1 ± 1.0	2.9 ± 4.6	0.2 (5.9%)	0.788	0.05	2.8 ± 1.0	2.7 ± 1.7	0.1 (2.6%)	0.853	0.05
Peak Acceleration IN (m/s^2^)	17.6 ± 5.3	16.6 ± 6.3	1.0 (6.3%)	0.247	0.18	19.0 ± 5.2	16.2 ± 5.8	2.8 (17.3%)	0.040	0.51
Peak Acceleration OUT (m/s^2^)	13.4 ± 4.4	12.3 ± 5.4	1.1 (8.7%)	0.165	0.22	12.7 ± 2.6	12.3 ± 5.1	0.4 (3.0%)	0.748	0.09
Cut Angle (°)	40.9 ± 29.7	55.2 ± 40.7	14.3 (25.9%)	0.013	0.40	36.9 ± 33.5	48.4 ± 36.17	11.5 (23.7%)	0.175	0.03

## Data Availability

The code will be made available by the authors upon reasonable request.

## Abbreviations

The following abbreviations are used in this manuscript:

ACL	Anterior cruciate ligament
KAM	Knee abduction moment
